# Evaluating Local Muscle Oxygen Saturation: Ischemic Preconditioning Protocols and the Myth of Overcompensation

**DOI:** 10.5114/jhk/194066

**Published:** 2024-12-06

**Authors:** Moacir Marocolo, Rhaí A. Arriel, Guilherme Guedes, Anderson Meireles, Michal Krzysztofik, Jakub Chycki, Adam Zajac, Hiago L. R. Souza

**Affiliations:** 1 Department of Biophysics and Physiology, Federal University of Juiz de Fora, Juiz de Fora, Minas Gerais, Brazil.; 2 Department of Training and Exercise Science, Faculty of Sport Science, Ruhr University Bochum, Bochum, Germany.; 3 Institute of Sport Sciences, The Jerzy Kukuczka Academy of Physical Education in Katowice, Katowice, Poland.

**Keywords:** blood flow, exercise, occlusion, ischemia, tissue saturation, pneumatic cuff

## Abstract

Ischemic preconditioning (IPC) is a promising strategy to enhance athletic performance and recovery by improving local muscle oxygen saturation. This study aimed to investigate the effects of different IPC protocols on muscle oxygenation in physically active healthy men. Thirty-four subjects were randomized into three groups and underwent four occlusion cycles of duration of three (IPC-3), five (IPC-5) or seven (IPC-7) min in only one limb. Near-infrared spectroscopy was used to assess oxygenated and deoxygenated hemoglobin levels, calculating total hemoglobin and oxygen saturation percentage (TSI%). Results showed significant improvements in muscle oxygenation following IPC, with variations in minimum, peak, and mean values across protocols. IPC-5 demonstrated the most consistent enhancements in oxygen saturation levels, with statistically significant differences observed in TSI% values during occlusion and reperfusion phases compared to IPC-3 (p < 0.05) and IPC-7 (p < 0.05), without supercompensation of TSI% during reperfusion phases compared to baseline. Specifically, IPC-5 induced the greatest increase in oxygenation in the contralateral limb compared to IPC-3 and IPC-7. These findings suggest that IPC, particularly the five-min protocol, effectively enhances local muscle oxygenation in non-occluded limbs, which may contribute to improved athletic performance and recovery, mainly under conditions where the exercise or testing does not directly involve cuffed limbs.

## Introduction

Intermittent blood flow occlusion/restriction and reperfusion interventions to optimize physical performance and/or recovery have aroused the interest of sports science researchers over the last 20 years (Arriel et al., 2020b; Bielec et al., 2013, 2023; Marocolo et al., 2018; Xiaolin et al., 2023). One type of this intervention, known as ischemic preconditioning (IPC), usually applied before or after an exercise or a test, involves applying pressure to a pneumatic cuff positioned on the proximal portion of either the upper or lower limbs, uni- or bilaterally, until complete occlusion of the blood flow is achieved, followed by reperfusion through cuff deflation (Marocolo et al., 2019).

IPC has promoted improvements in athletic performance (Arriel et al., 2020a; Marocolo et al., 2019) and recovery (Arriel et al., 2018), across different types and duration of exercise or tests. It is purported that one of the mechanisms for the enhancement effects of IPC on exercise performance is mediated through specific triggers in humoral pathways (Stokfisz et al., 2017) which facilitate vasodilatation (Cocking et al., 2018; Enko et al., 2011) by the release of vasoactive compounds (Volga Fernandes et al., 2022), improvements in the blood flow (Mota et al., 2020) and consequently, an increase in oxygen delivery by altering oxy- and deoxygenation (Kjeld et al., 2014; Tanaka et al., 2016). These changes in the blood flow and oxygen levels (Jackson et al., 2023; Mota et al., 2020) may facilitate more favorable outcomes in modalities reliant on the cardiovascular system and oxidative pathways, as opposed to high-intensity, short-duration exercises (e.g., jumps and sprints).

Nevertheless, the heightened blood flow observed during the hyperemia phase following cuff release typically endures for no more than 100 s (Mota et al., 2020), after which a consistent pattern of local muscle oxygenation is observed (Bopp et al., 2014; Jackson et al., 2023). Additionally, artery vasodilation in the contralateral limb (Enko et al., 2011), attenuation of artery-flow mediated dilation (Bailey et al., 2012) and an augmentation in vascular conductance (Rytter et al., 2020) have been reported post-cuff intervention, although measurements of the blood flow or muscle oxygenation were not consistently provided. In this context, measuring local muscle oxygenation may provide direct insights into the physiological adaptations and efficacy of IPC. It remains undemonstrated that IPC effects could be attributed to the myth of overcompensation in tissue oxygenation and mitochondrial function, which are key indicators of muscle health and recovery (Barstow, 2019). This measurement is particularly relevant in clinical and athletic settings, where optimizing tissue perfusion and oxygen delivery can enhance performance and recovery (Crisafulli et al., 2011; de Groot et al., 2010). Furthermore, understanding the dynamics of muscle oxygenation during and after IPC can refine the protocols for various applications, ensuring maximal benefits and minimal adverse effects (Kido et al., 2015).

In this context, near-infrared spectroscopy (NIRS) emerges as a non-invasive technological method capable of estimating local muscle oxygenation by assessing variables such as total hemoglobin (THb) and oxygen local saturation (SmO_2_), often expressed as a percentage of the tissue saturation index (TSI%) (Feldmann et al., 2019). NIRS has been utilized to measure the TSI during the reperfusion phase in IPC intervention, following short (1.5 min) or longer (5 min) cuff occlusion (Jones et al., 2014), as well as during a resistance exercise protocol (Meireles et al., 2023). However, there remains a dearth of continuous evaluation before, during and after IPC protocols in the existing literature.

Such investigation could be instrumental in comprehending the tissue oxygenation response induced by IPC and refining optimal protocols to enhance exercise performance and/or facilitate recovery. Hence, this study aimed to verify the possible increase in the tissue saturation index at rest, and after different IPC protocols. Our hypothesis was that an extended occlusion duration would induce a more pronounced compensatory response in local muscle oxygenation.

## Methods

### Participants

A priori sample estimation (G*Power 3.1.9.2, Heinrich-Heine Universität Düsseldorf, Düsseldorf, Germany, (www.gpower.hhu.de) using a repeated measures ANOVA, within-between interactions with a moderate effect size (f = 0.25), α = 0.05, power (1-β) = 0.8 and a correlation of 0.5 indicated a minimum sample size of 30 subjects (i.e., 10 per group). We therefore included in the study thirty-four physically active healthy men (Table 1). Eligibility criteria stipulated men aged between 18 and 40 years who engaged in regular physical activity at least twice a week. The criteria also included non-smokers, absence of any cardiovascular or metabolic disease, systemic normotension (<140/90 mmHg), no use or history of anabolic steroids, drugs, or medications with potential effects on the cardiovascular system or physical performance (self-reported), and no recent musculoskeletal injury.

**Table 1:** Anthropometric and demographic characteristics of the study’s participants.

Characteristics	IPC-3 (n = 12)	IPC-5 (n = 12)	IPC-7 (n = 10)	*p* value
Age (years)	21.2 ± 5.1	21.4 ± 5.1	23.1 ± 5.7	0.67
Body height (m)	1.71 ± 0.1	1.70 ± 0.1	1.76 ± 0.1	0.49
Body mass (kg)	71.1 ± 11.3	71.3 ± 8.5	76.1 ± 12.8	0.96
Thigh circumference (cm)	48.7 ± 7.4	47.2 ± 6.8	47.5 ± 5.8	0.83
Thigh skinfold (mm)	12.7 ± 4.5	11.8 ± 2.4	11.4 ± 2.6	0.66
SBP (mmHg)	128.0 ± 7.2	125.0 ± 5.5	126.0 ± 5.0	0.79
DBP (mmHg)	83.0 ± 3.6	83.0 ± 4.0	80.0 ± 3.2	0.85
AOP (mmHg)	175.2 ± 11.0	173.7 ± 10.9	168.8 ± 10.0	0.36
Training experience (years)	4.1 ± 2.2	3.9 ± 2.3	4.4 ± 2.1	0.71
Training (hours/week)	3.6 ± 1.8	3.9 ± 2.0	3.4 ± 2.1	0.75

Data are presented as mean ± SD. IPC: ischemic preconditioning; SBP: systolic blood pressure; DBP: diastolic blood pressure; AOP: arterial occlusion pressure

### Experimental Design and Procedures

After the recruitment of participants, by E-mail, phone or personally, the first laboratory visit included anthropometric measurements (body height, body mass, circumferences, skinfold thickness) and screening questionnaires. Following a 5-min rest interval in a supine position, systolic (SBP) and diastolic (DBP) arterial blood pressures were measured. Following an additional 10-min rest interval, arterial occlusion pressure (AOP) was measured. At the second laboratory visit, using a randomized parallel-participant design, participants were allocated into one of the three IPC protocol conditions: IPC-3, IPC-5 or IPC-7. Randomization was conducted via a random number sequence generated online (www.randomization.com). Local oxygen saturation (SmO_2_) was measured using three portable NIRS sensors under a resting condition. Measurements were taken before, during, and after the IPC protocols.

**Figure 1:**
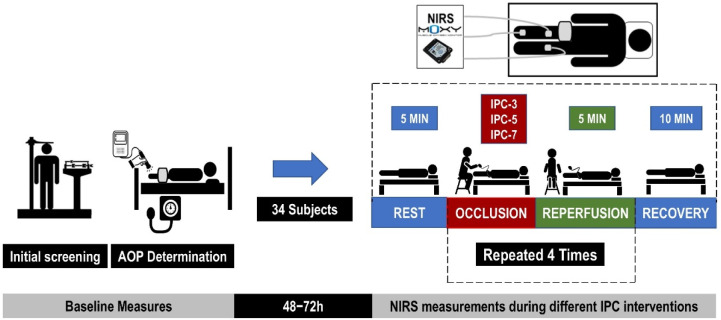
Experimental design of the study. Subjects were randomly included in one of the three protocols: IPC-3, IPC-5 or IPC-7, lasting three, five, or seven min of occlusion, respectively. AOP: arterial occlusion pressure; IPC: ischemic preconditioning; NIRS: near-infrared spectroscopy

All data were collected in a controlled environment (23.5 ± 1.0°C; relative humidity: 68.2 ± 5.30%) during the same period of the day (09:00–11:00 a.m.), aimed at minimizing circadian influences (Baxter and Ray, 2020; Teo et al., 2011). Participants were instructed to maintain a regular diet and abstain from moderate or intense physical exercise for 48 h prior to testing, as well as from caffeine consumption within 12 h preceding the experiment. The experimental procedure received approval from the Institutional Review Board of the Federal University of Juiz de Fora (approval code: 4.120.625; date of approval: 29 June 2020) and was conducted in accordance with the Declaration of Helsinki. All subjects gave written informed consent prior to participation. Figure 1 shows the experimental design of the study.

### Arterial Occlusion Pressure Determination

After 5 min of supine rest, a pneumatic cuff (96 cm in length x 13 cm in width; Komprimeter Riester^®^, Jungingen, Germany) was placed on the proximal region of the right thigh and inflated until reaching the individual SBP, followed by increments of 10 mmHg until the blood flow was no longer detected (de Oliveira et al., 2024). The pulse in the limb was detected using a hand-held bidirectional vascular Doppler positioned at the anterior tibial artery. Audible signals from the Doppler probe indicated the presence of pulse. AOP was recorded when no audible signal was detected. Previous evidence has demonstrated the high reliability of the hand-held Doppler in measuring AOP (Vehrs et al., 2023, 2024)

### IPC Protocols

The same pneumatic cuff (96 cm in length x 13 cm in width; Komprimeter Riester^®^, Jungingen, Germany) was positioned at the proximal portion of the right thigh and inflated until 20 mmHg above the individual’s AOP. Each occlusion cycle was alternated with 5 min of reperfusion at 0 mmHg cuff pressure. To ensure proper occlusion of the blood flow during the protocol, a hand-held bidirectional Doppler probe was placed on the posterior tibial artery (Loenneke et al., 2013; Oranchuk et al., 2020). The contralateral limb was used as control with no cuff administered.

Each group underwent four cycles of cuff occlusion lasting 3 min (IPC-3), 5 min (IPC-5) or 7 min (IPC-7), respectively, followed by 5 min of blood flow reperfusion in all three protocols. According to previous research, the most commonly used protocol consisted of four cycles of 5-min occlusion-reperfusion (Marocolo et al., 2019). Therefore, we explored different occlusion time within this protocol.

### Near-Infrared Spectroscopy (NIRS)

Local oxygen saturation (SmO_2_) was assessed using three portable NIRS sensors (Moxy, Fortiori Design LLC, Hutchinson, USA) before, during and after cuff intervention (Figure 1). The NIRS device operates by sequentially sending light waves (630–850 nm) from four light-emitting diodes into the tissue beneath it and recording the amount of scattered light returned to two detectors positioned 12.5 mm and 25 mm from the light source (Crum et al., 2017). The scattered light is analyzed using an algorithm that integrates a tissue light propagation model with the Beer-Lambert law to assess the amount of light absorbed at wavelengths corresponding to oxygenated and deoxygenated hemoglobin (Hb) (Crum et al., 2017; Feldmann et al., 2019). This enables the calculation of total hemoglobin (THb) present beneath the device, as well as the percentage of hemoglobin saturated with oxygen (SmO_2_) (Feldmann et al., 2019), expressed in this study as the TSI%. For the experimental procedure, the skin was cleaned with an alcohol pad, and the sensors were inserted in silicone cases and attached to the skin with single-sided micropore tape. Black bandages covered the device to eliminate background light. Three NIRS sensors were placed at different subjects’ sites: the first sensor was placed on the vastus medialis (VM) muscle, one third of the distance from the top of the patella to the greater trochanter, and the second sensor was placed on the medial portion of the gastrocnemius muscle, both on the cuffed limb under intervention. The last sensor was placed on the contralateral limb at the same corresponding position of the VM. The position was marked with a permanent pen for the subsequent visit. Skinfold thickness was measured at the site of NIRS application using a Lange skinfold caliper (Beta Technology Inc., Houston, TX, USA) during the familiarization session. The validity and reliability of the NIRS sensor (SROC: r = 0.842–0.993, ICC: r = 0.773–0.992, *p* < 0.01) have been confirmed in previous studies (Crum et al., 2017; Feldmann et al., 2019).

### Pain Scale

As we have already used the lowest cuff pressure necessary for occlusion, the assessment of pain in relation to time could be another relevant factor when applying an IPC protocol. To evaluate whether the cuff administration time causes greater perceptions of pain, the pain perception was assessed during the IPC protocol using a visual analogue scale (0–10 score), where 0 indicated “no pain” and 10 indicated “unbearable pain”. The following instructions were used: “Select a single number that best represents the pain intensity felt during this intervention” (Ferreira-Valente et al., 2011).

### Data Processing

The time-course of local muscle oxygenation (TSI%) using NIRS sensors was recorded from the non-occluded thigh (Control), the occluded thigh (Thigh) and the occluded leg (Leg). Data were divided into sections for analysis: baseline (5 min), occlusion and reperfusion phases, and recovery (5 min and 10 min after the last reperfusion phase) and the mean values were used for statistical comparisons. Additionally, minimum, peak and mean TSI% values for each NIRS-time point were registered.

The analysis of IPC intervention intervals was divided into two cases: A) within protocols, where the three protocols were compared in relation to different cycles and recovery to baseline. This analysis was conducted individually for each NIRS sensor data; and B) within sensors, where values were compared during non-cuff periods (basal and recovery). This analysis was conducted for the three proposed protocols.

### Statistical Analysis

Shapiro-Wilk and Levene’s tests were performed to verify normality and homoscedasticity of the TSI% data, respectively. Separate three-way mixed-model ANOVA was conducted to analyze effects of the protocol (IPC-3, IPC-5 and IPC-7), NIRS sensors (thigh, leg and control) and time (baseline, IPC cycles, and recovery) on minimum, peak and mean TSI% values. When necessary, Bonferroni post hoc correction was conducted for pairwise comparisons. A Friedman and Kruskal-Wallis tests were conducted to compare perceived pain values within-between IPC protocols throughout cuff occlusion cycles. Effect size (ES) was calculated using Cohen’s *d*, where < 0.2 = trivial, > 0.2–0.6 = small, > 0.6–1.2 = moderate, > 1.2–2.0 = large and > 2.0–4.0 = very large (Batterham and Hopkins, 2006). The level of significance adopted was *p* < 0.05 and GraphPad Prism® Software (Version 8.0, San Diego, CA, USA) was used for the analysis.

## Results

### TSI% during IPC Protocols and Perceived Pain

**Figure 2:**
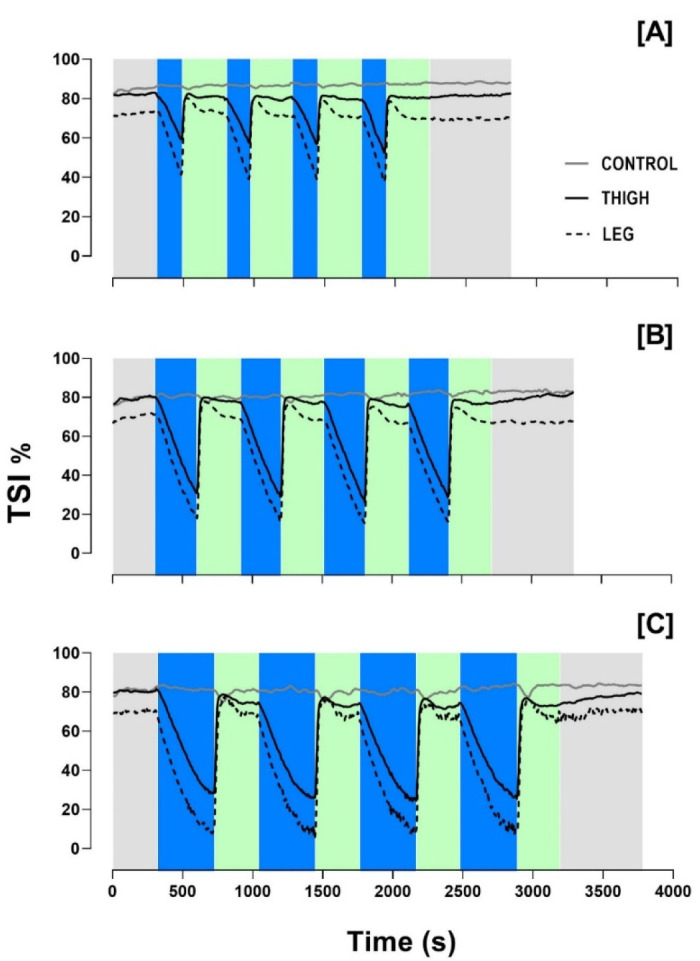
Tissue saturation index values (expressed as a percentage, TSI%) measured with NIRS sensors in non-occluded (Control) and occluded (Thigh and Leg) limbs are depicted in [A] for IPC-3, [B] for IPC-5 and [C] for IPC-7. Gray areas indicate baseline and recovery sections; blue areas represent occlusion phases; green areas represent reperfusion phases.

Variations in the TSI% measured by NIRS sensors during IPC protocols are depicted in Figure 2. Due to technical issues with the portable NIRS sensors, the TSI% was recorded in 8, 10, and 7 subjects for the IPC-3, IPC-5 and IPC-7 protocols, respectively. There were significant decreases and increases in the TSI% during occlusion and reperfusion phases in all IPC protocols (*p* < 0.05). The magnitude of decreases in the TSI% were directly related to the time of occlusion, with IPC-5 and IPC-7 promoting the lowest values for thigh and leg NIRS sensors compared to IPC-3. For the control limb, no differences in TSI% values were found among IPC protocols during occlusion phases. During reperfusion phases, no difference in TSI% was found among IPC protocols. Table 2 presents the minimum and mean values of the TSI% during occlusion, as well as the mean and peak values during reperfusion cycles, for all IPC protocols, according to the sections previously established in Figure 2.

**Table 2:** TSI% values during and throughout recovery time after the application of IPC protocols.

			TSI% during IPC protocols application									
			IPC-3			IPC-5			IPC-7			
			Thigh	Control	Leg	Thigh	Control	Leg		Thigh	Control	Leg	
**1^st^ cycle**	OCC	Min	61.5 ± 9.7	85.4 ± 8.0	40.9 ± 17.1	28.9 ± 16.2*	78.0 ± 6.6	17.9 ± 16.5*		25.5 ± 18.2*	78.6 ± 10.0	6.3 ± 7.4*	
		Mean	74.1 ± 6.5	86.5 ± 7.2	59.4 ± 14.8	53.6 ± 9.6*	80.9 ± 6.2	43.6 ± 12.7*		50.0 ± 11.8*	81.9 ± 7.3	29.5 ± 8.2*	
	REP	Peak	84.4 ± 5.6	87.6 ± 7.5	81.6 ± 7.7	80.6 ± 5.6	82.3 ± 6.4	79.1 ± 6.6		82.8 ± 3.3	82.8 ± 7.2	79.5 ± 10.5	
		Mean	81.6 ± 6.8	86.2 ± 8.5	74.0 ± 12.4	75.0 ± 5.4	79.6 ± 7.4	69.8 ± 7.8		77.3 ± 4.3	79.6 ± 8.6	69.0 ± 8.3	
**2^nd^ cycle**	OCC	Min	60.5 ± 11.4	85.6 ± 8.4	38.7 ± 17.6	25.1 ± 15.5*	77.7 ± 8.6	15.5 ± 14.9*		22.7 ± 19.8*	77.8 ± 10.8	5.6 ± 7.0*	
		Mean	72.9 ± 6.6	86.8 ± 7.5	57.1 ± 15.7	52.4 ± 7.6*	80.4 ± 7.7	44.1 ± 10.0*		44.0 ± 15.3*	81.0 ± 8.7	27.5 ± 6.8*	
	REP	Peak	83.6 ± 7.4	88.5 ± 5.6	80.4 ± 7.5	80.7 ± 4.8	82.6 ± 7.5	78.1 ± 6.7		82.2 ± 4.1	82.7 ± 7.6	79.8 ± 11.7	
		Mean	80.6 ± 8.3	86.4 ± 8.3	72.5 ± 12.0	75.5 ± 5.0	79.9 ± 8.3	69.5 ± 6.7		76.4 ± 5.2	79.2 ± 9.2	68.1 ± 7.5	
**3^rd^ cycle**	OCC	Min	59.4 ± 9.3	86.2 ± 7.6	38.7 ± 16.9	23.7 ± 15.6*	79.7 ± 8.6	14.7 ± 12.9*		20.9 ± 21.1*	78.1 ± 10.4	5.7 ± 7.9*	
		Mean	72.3 ± 6.6	87.3 ± 6.8	57.1 ± 14.4	48.9 ± 9.0*	81.8 ± 7.5	40.3 ± 8.2*		43.9 ± 15.4*	80.3 ± 9.5	28.2 ± 6.1*	
	REP	Peak	83.6 ± 7.0	88.4 ± 6.4	80.0 ± 6.9	80.4 ± 6.2	83.8 ± 7.0	77.2 ± 7.3		83.2 ± 4.6	83.1 ± 7.2	78.6 ± 11.1	
		Mean	80.5 ± 8.0	86.8 ± 8.1	72.0 ± 11.6	73.7 ± 5.4	80.6 ± 8.9	68.2 ± 7.3		76.6 ± 5.1	79.5 ± 8.8	66.8 ± 6.9	
**4^th^ cycle**	OCC	Min	54.0 ± 13.7	86.4 ± 8.6	37.7 ± 17.6	23.4 ± 13.7*	79.8 ± 9.5	15.6 ± 11.4*		22.6 ± 23.5*	79.4 ± 9.1	5.9 ± 7.7*	
		Mean	68.4 ± 9.7	87.3 ± 7.6	54.5 ± 14.7	47.4 ± 7.4*	82.6 ± 7.8	39.5 ± 6.1*		45.4 ± 17.5*	82.5 ± 7.2	28.7 ± 5.1*	
	REP	Peak	83.3 ± 7.6	89.0 ± 6.0	79.4 ± 7.3	79.7 ± 5.6	84.5 ± 7.2	77.6 ± 7.3		83.4 ± 4.9	84.6 ± 6.0	79.2 ± 11.5	
		Mean	80.7 ± 8.3	87.5 ± 7.6	71.0 ± 11.8	73.3 ± 5.3	82.3 ± 8.2	67.8 ± 6.6		77.0 ± 6.6	82.2 ± 7.4	67.4 ± 7.1	
			**TSI% at baseline and throughout recovery time after IPC protocols application**									
			IPC-3			IPC-5			IPC-7			
			Thigh	Control	Leg	Thigh	Control	Leg		Thigh	Control	Leg	
**Baseline**		Min	80.8 ± 4.1	81.6 ± 12.7	68.3 ± 15.6	73.1 ± 7.8	73.0 ± 11.3	64.7 ± 12.8		78.6 ± 6.4	76.3 ± 11.4	65.2 ± 10.1	
		Peak	85.1 ± 4.0	86.4 ± 9.1	75.6 ± 14.3	80.9 ± 5.2	82.1 ± 6.4	73.1 ± 10.6		84.0 ± 3.2	84.3 ± 5.5	74.2 ± 11.8	
		Mean	83.3 ± 4.0	84.6 ± 10.6	72.3 ± 15.1	78.3 ± 5.4	78.9 ± 7.5	70.1 ± 11.6		82.3 ± 4.3	81.0 ± 7.2	70.0 ± 10.5	
**Recovery 5 min**		Min	80.6 ± 8.6	86.6 ± 8.5	67.0 ± 15.7	72.9 ± 6.8	81.1 ± 9.8**^#^**	64.1 ± 9.1		77.4 ± 6.9	80.8 ± 7.7	62.1 ± 7.9	
		Peak	84.2 ± 6.7	89.0 ± 6.2	71.9 ± 13.5	79.7 ± 5.6	85.0 ± 7.3**^#^**	70.0 ± 8.2		82.7 ± 4.9	85.8 ± 5.9	74.8 ± 12.1	
		Mean	82.5 ± 7.6	87.8 ± 7.6	68.3 ± 14.4	76.5 ± 6.0	83.1 ± 8.5**^#^**	67.3 ± 8.6		79.9 ± 5.9	83.3 ± 6.4	68.3 ± 8.8	
**Recovery 10 min**		Min	82.5 ± 6.6	86.8 ± 8.4	67.3 ± 16.8	76.3 ± 7.8	80.5 ± 12.0**^#^**	63.8 ± 9.4		79.9 ± 6.0	81.9 ± 6.9	64.4 ± 8.6	
		Peak	84.8 ± 6.3	89.3 ± 5.8**^#^**	72.3 ± 14.3	82.2 ± 6.7	85.3 ± 7.5**^#^**	70.8 ± 8.9		83.4 ± 4.9	84.9 ± 5.8	74.7 ± 11.2	
		Mean	83.7 ± 6.4	88.1 ± 7.2	69.7 ± 15.5	79.2 ± 7.1	83.0 ± 9.4**^#^**	67.2 ± 9.1		81.9 ± 5.3	83.6 ± 6.3	70.5 ± 9.4	

Data are expressed as mean ± SD; OCC: occlusion phase; REP: reperfusion phase; Min: minimum; IPC-3 (n = 8), IPC-5 (n = 10), IPC-7 (n = 7). * significant difference to IPC-3 within the same limb NIRS sensor and cycle (p < 0.05); # significant difference to baseline within the same limb, protocol and measure (i.e., min, peak and mean) (p < 0.05)

**Figure 3:**
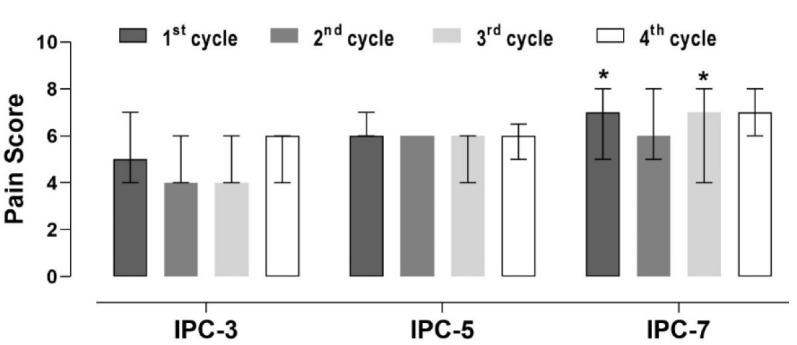
Perceived pain values during the application of IPC protocols. Data are median (interquartile interval); 1^st^ cycle, 2^nd^ cycle, 3^rd^ cycle, 4^th^ cycle; n = 11 per protocol. * significant difference to IPC-3 within the same cycle (p < 0.05)

Pain perception throughout the IPC application was higher in IPC-7 after the first and third cuff occlusion cycles compared to IPC-3 (Figure 3). No other significant differences in pain perception were found among IPC protocols (*p* > 0.05).

### TSI% at Baseline and Throughout the Recovery after IPC Protocols Application

Table 2 shows the minimum, mean and peak TSI% values at baseline and during the 5^th^ and the 10^th^ min after IPC protocols. The TSI% increase was found in minimum, peak and mean values from baseline to the 5^th^ and the 10^th^ min after IPC application in the control limb for the IPC-5 protocol, as depicted in Table 3. Additionally, an increase in TSI% peak value was observed 10 min after IPC application in the control limb for the IPC-3 protocol compared to baseline (absolute change of 2.9 ± 3.9, *p* = 0.046, 95%CI = −0.3–6.2; ES = 0.39 [small], 95% CI for ES = −0.33–1.10). The thigh and the control limb presented higher TSI% minimum, peak and mean values compared to the leg at the baseline, at the 5^th^ and the 10^th^ min after IPC application for all protocols. No significant differences were found among IPC protocols or between the thigh and the control limb (*p* > 0.05).

**Table 3:** The absolute changes in minimum, peak and mean TSI% values from the baseline to the 5^th^ and the 10^th^ min after IPC-5 protocol application in the control limb.

	TSI% change (mean ± SD)	*p*_value_	95% CI	ES (magnitude)	95% CI for ES
**Minimum**					
Baseline to the 5**^th^** min	8.1 ± 9.1	0.010	1.6–14.6	0.77 (moderate)	0.06–1.39
Baseline to the 10**^th^** min	7.5 ± 9.5	0.017	0.7–14.3	0.64 (moderate)	−0.04–1.26
**Peak**					
Baseline to the 5**^th^** min	2.9 ± 2.9	0.027	0.8–4.9	0.42 (small)	−0.22–1.05
Baseline to the 10**^th^** min	3.2 ± 3.1	0.013	1.0–5.4	0.46 (small)	−0.19–1.09
**Mean**					
Baseline to the 5**^th^** min	4.2 ± 4.3	0.010	1.1–7.2	0.52 (small)	−0.14–1.15
Baseline to the 10**^th^** min	4.1 ± 4.8	0.014	0.7–7.5	0.49 (small)	−0.17–1.11

ES: effect size; CI: confidence interval

## Discussion

This study investigated the effects of different IPC protocols on the TSI% and perceived pain. Specifically, the IPC protocols involved four cycles of three, five or seven min of cuff occlusion, each interspersed with 5 min of reperfusion. The primary findings indicate significant variations in the TSI% during the occlusion and reperfusion phases among the IPC protocols, with IPC-5 and IPC-7 inducing the most pronounced decreases in the TSI% during occlusion. Additionally, higher perceived pain during occlusion phases was found in IPC-7 compared to IPC-3.

The decrease in the TSI% during occlusion was more pronounced in IPC-5 and IPC-7 compared to IPC-3, which can be attributed to the longer duration of occlusion phases. These findings align with previous research demonstrating that longer occlusion times lead to greater reductions in tissue oxygenation due to sustained ischemia (Kooijman et al., 2008). All subjects in our study were healthy, physically active males, and no outlier responses were identified. However, during reperfusion, TSI% values returned to baseline levels across all protocols, indicating effective restoration of tissue oxygenation post-occlusion, but not overcompensation for this variable. This is consistent with prior research demonstrating rapid reoxygenation following the release of occlusion, which is crucial for inducing protective ischemic preconditioning effects (Crisafulli et al., 2011). These findings suggest that occlusion time duration does not exhibit a linear relationship with reoxygenation, highlighting the potential preference for shorter protocols, particularly in sports scenarios such as halftime or congested match schedules, which would be in line with no more than 5 min of cuff-occlusion during IPC protocols.

Although IPC protocols did not cause TSI% overcompensation during reperfusion phases (i.e., after each occlusion cycle and after the end of the IPC protocol) in the occluded limb, the IPC-5 protocol elicited a greater increase in oxygenation in the contralateral non-occluded (Control) limb compared to the IPC-3 and IPC-7 protocols during the 10^th^ min measured after the end of the protocol (Table 2, lower part, recovery 5 min and 10 min), which can perhaps be explained by the need for optimal occlusion duration to cause this tissue saturation overcompensation. IPC has already promoted contralateral upper limb artery vasodilation both at rest (Enko et al., 2011) and during 30 min of the rhythmic handgrip test (Cocking et al., 2018). This increase may suggest that the effects of IPC on the TSI% have a greater systemic impact compared to a localized effect.

Based on this increase in muscle oxygenation together with the possible raise in the blood flow, it could be suggested using IPC on limbs not directly involved in the test or exercise, which is called the remote IPC [RIPC] (Marocolo et al., 2016). For instance, applying a cuff to the upper limbs might be beneficial when considering a jump test. Most studies on IPC and performance have traditionally administered the cuff to the lower limbs and conducted tests such as running, cycling or resistance exercises targeting the same region, which is not in line with our findings. Additionally, exploring IPC in this manner could broaden its application across various sports and activities, offering a better consistency for the use of the RIPC strategy to enhance athletic performance without direct preconditioning of the primary muscles engaged in the activity.

The observed increases in the TSI% from baseline to recovery periods in IPC-5 in the control limb, indicate a beneficial effect of the protocol in enhancing post-ischemic tissue oxygenation. Such increases suggest improved microcirculatory function and tissue perfusion following IPC application, consistent with findings from other studies that reported enhanced endothelial function and vascular reactivity post-IPC (Bailey et al., 2012; Jones et al., 2014). Also, the possible redirection of the blood flow to the non-occluded limb may contribute to this increase. Furthermore, we could not link the more pronounced hypoxic condition in the leg compared to the thigh with significant increases in the TSI response. This fact could be due to collateral micro- and circulation of the thigh that did not occur at the leg.

The higher pain perception reported during IPC-7 suggests a potential limitation of using extended occlusion periods. Pain associated with prolonged ischemia might limit the practicality and adherence to such protocols in clinical and athletic settings. These findings are in line with previous evidence that has documented increased discomfort with longer ischemic duration (Turnes et al., 2018) or higher applied pressures (Meireles et al., 2023; Sharma et al., 2014). Furthermore, since the longer IPC-7 protocol did not show additional benefits over the IPC-5 protocol, and extended occlusion time increased pain and discomfort, IPC protocols with no longer than 5-min occlusion periods should be used. This adjustment could improve the overall feasibility of the protocol. Also, the use of the minimum cuff width of 9 cm and 13 cm on the arm or the thigh, as we have applied in this study, would ensure a lower arterial occlusion pressure and lower pain for the subjects.

Since we did not conduct a direct assessment of the impact of the IPC protocol on changes in athletic performance it is not possible to be certain whether changes in the TSI% would be associated with general physical performance. Additionally, reperfusion was examined until the 10^th^ min after IPC, at rest, what limits the extrapolation of these results to extended time-frames.

## Conclusions

Our findings indicate that the IPC-5 protocol effectively induces significant ischemic preconditioning effects while maintaining tolerable pain levels. This evidence underscores the potential of remote IPC-5 min (control limb) in improving tissue oxygen saturation (TSI%) during the recovery phase, typically the starting point of an exercise test.
